# Protocol for a randomised trial of early kangaroo mother care compared to standard care on survival of pre-stabilised preterm neonates in The Gambia (eKMC)

**DOI:** 10.1186/s13063-020-4149-y

**Published:** 2020-03-06

**Authors:** Helen Brotherton, Abdou Gai, Cally J. Tann, Ahmadou Lamin Samateh, Anna C. Seale, Syed M. A. Zaman, Simon Cousens, Anna Roca, Joy E. Lawn

**Affiliations:** 1 Faculty of Epidemiology and Population Health, and MARCH Centre, London School of Hygiene & Tropical Medicine (LSHTM), Keppel Street, London, UK; 2 MRC Unit The Gambia at LSHTM, Atlantic Road, Fajara, The Gambia; 3 Department of Medical Paediatrics, Royal Hospital for Sick Children, Edinburgh, UK; 4 MRC/UVRI & LSHTM Uganda Research Unit, Plot 51-59 Nakiwogo Road, Entebbe, Uganda; 5 Neonatal Medicine, University College London Hospitals NHS Trust, 235 Euston Rd, London, UK; 6 Ministry of Health and Social Welfare, Gambia Government, Banjul, The Gambia; 7 Education Department, Liverpool School of Tropical Medicine, Pembroke Place, Liverpool, UK

**Keywords:** Preterm, Neonate, Kangaroo care, Kangaroo mother care, Skin-to-skin contact, Survival, Infection, Randomised controlled trial, Pragmatic

## Abstract

**Background:**

Complications of preterm birth cause more than 1 million deaths each year, mostly within the first day after birth (47%) and before full post-natal stabilisation. Kangaroo mother care (KMC), provided as continuous skin-to-skin contact for 18 h per day to fully stabilised neonates ≤ 2000 g, reduces mortality by 36–51% at discharge or term-corrected age compared with incubator care. The mortality effect of starting continuous KMC before stabilisation is a priority evidence gap, which we aim to investigate in the eKMC trial, with a secondary aim of understanding mechanisms, particularly for infection prevention.

**Methods:**

We will conduct a single-site, non-blinded, individually randomised, controlled trial comparing two parallel groups to either early (within 24 h of admission) continuous KMC or standard care on incubator or radiant heater with KMC when clinically stable at > 24 h of admission. Eligible neonates (*n* = 392) are hospitalised singletons or twins < 2000 g and 1–24 h old at screening who are mild to moderately unstable as per a trial definition using cardio-respiratory parameters. Randomisation is stratified by weight category (< 1200 g; ≥ 1200 g) and in random permuted blocks of varying sizes with allocation of twins to the same arm. Participants are followed up to 28 ± 5 days of age with regular inpatient assessments plus criteria-led review in the event of clinical deterioration. The primary outcome is all-cause neonatal mortality by age 28 days. Secondary outcomes include the time to death, cardio-respiratory stability, hypothermia, exclusive breastfeeding at discharge, weight gain at age 28 days, clinically suspected infection (age 3 to 28 days), intestinal carriage of extended-spectrum beta-lactamase producing (ESBL) *Klebsiella pneumoniae* (age 28 days), and duration of the hospital stay. Intention-to-treat analysis will be applied for all outcomes, adjusting for twin gestation.

**Discussion:**

This is one of the first clinical trials to examine the KMC mortality effect in a pre-stabilised preterm population. Our findings will contribute to the global evidence base in addition to providing insights into the infection prevention mechanisms and safety of using this established intervention for the most vulnerable neonatal population.

**Trial registration:**

ClinicalTrials.gov NCT03555981. Submitted 8 May 2018 and registered 14 June 2018. Prospectively registered.

## Background

Every year an estimated 14.8 million neonates are born preterm (< 37 completed weeks of gestation), of which > 80% are in Asia or Sub-Saharan Africa [1], and more than 1 million die due to complications of prematurity [2]. An estimated 47% of all prematurity-related deaths in resource-limited settings occur within the first day after birth [3] before post-natal stabilisation is complete. This is the critical period in which to target interventions to improve preterm survival and accelerate progress toward the Sustainable Development Goal (SDG) target 3.2 for neonatal mortality reduction. More than 40 countries, many in sub-Saharan Africa, need to more than double their current progress to meet the target by 2030 [4].

Kangaroo mother care (KMC) is an evidence-based package recommended as standard care for all clinically *stable* (pre-stabilised) neonates < 2000 g [5], which is the proxy weight used in previous KMC trials as an indicator for preterm birth [6]. Described in Colombia four decades ago, KMC has since been widely adopted as a cornerstone of neonatal care. The key component is prolonged, skin-to-skin contact between neonate and caregiver, facilitating exclusive breastmilk feeding and shorter hospital stay [7].

Clinical stability is variably defined in previous KMC trials with no standardised WHO definition or validated clinical model for resource-limited settings. In neonates < 2000 g who have completed stabilisation or post-natal transition, continuous KMC (aiming for > 18 h/day) reduces mortality at discharge or 40 weeks post-menstrual age by 36–51% [6, 8, 9] compared to incubator care, with the mortality effect observed only in resource-limited settings [6]. However, an evidence gap exists for neonates yet to complete stabilisation, who have greatest risk of death or adverse outcome [6]. In 20 trials that assessed mortality at latest follow-up and were included in three systematic reviews [6, 8, 9], KMC was initiated at an average age ≤ 4 days in seven trials, with only one RCT starting continuous KMC in pre-stabilised neonates within 24 h after birth [10]. This Ethiopian trial reported a 40% reduction in mortality (RR = 0.57, 95% CI 0.33–1.00, *p* < 0.05) but more than half of the unstable neonates were excluded, and the eligibility criteria were unclear, leading to high risk of bias [6, 10].

KMC is a safe intervention for unstable neonates in resource-rich settings with intensive monitoring [11], but the safety profile in a context of less close clinical monitoring is not established [6] and warrants further scrutiny.

KMC works through multiple pathways, many mediated by skin-to-skin contact [12], including thermal control [6], neuro-endocrine mechanisms involving oxytocin release in both mother and neonate [12], reduced cortisol and stress response [13], cardio-respiratory stabilisation [14], enhanced breast milk production [6] and empowerment of the KMC provider in caring for their baby. Alterations in the neonatal microbiome with intermittent KMC have also been reported [15] and warrant further exploration to understand the infection prevention effects of KMC. The relevance and relative contribution of these mechanisms for KMC in pre-stabilised neonates are unknown, particularly for infection prevention outcomes, which is an evidence gap for all preterm neonates.

The eKMC trial aims to investigate continuous KMC in pre-stabilised neonates < 2000 g in a Gambian health facility setting. A secondary aim is to explore potential underlying mechanisms of KMC in this high-risk population.

### Objectives

The primary objective of the eKMC trial is to assess the effect of early continuous KMC on the survival of pre-stabilised preterm neonates.

### Secondary objectives

Secondary objectives include the following:

1. Assess the effect of early continuous KMC on other important clinical outcomes (growth, late-onset infections and duration of hospital stay)
2. Evaluate the safety of providing early continuous KMC to pre-stabilised preterm neonates in a resource limited facility setting
3. Explore possible mechanisms for hypothesised beneficial effects of early continuous KMC in pre-stabilised preterm neonates, focusing on infection prevention

## Methods/design

This article has been prepared according to the Standard Protocol Items: Recommendations for Interventional Trials (SPIRIT) statement (Additional file 1) [16].

### Study design

This single-site, pragmatic, non-blinded, individually randomised superiority trial compares two parallel groups managed with either continuous KMC started within 24 h of hospital admission or standard care with intermittent or continuous KMC when clinically stable > 24 h after admission. The unit of randomisation is the mother in a 1:1 ratio with twin participants randomised to the same arm.

### Study setting and context

Recruitment began on 23 May 2018 and is ongoing at the neonatal unit of Edward Francis Small Teaching Hospital (EFSTH), the main neonatal referral unit in The Gambia, with research support from the MRC Unit of Gambia at London School of Hygiene & Tropical Medicine (MRCG at LSHTM).

The Gambia is the smallest country in mainland Africa, with a population of 2.1 million, and it is ranked 174/189 on the Human Development Index (2017) [17]. Neonatal mortality declined from 49 to 26 per 1000 live births between 1990 and 2018, respectively [18], with 12–14% of Gambian neonates born preterm [1, 19] and 29% of neonatal deaths attributed to complications of prematurity [3].

A quarter (26%) of the 1400 annual neonatal admissions to EFSTH are due to prematurity [20], and the neonatal case fatality rate is 38%, with the highest rate (58%) occurring amongst neonates born < 1500 g [20]. Both in-born (born at the EFSTH maternity unit) and out-born (born at another health facility or home) neonates are admitted from a mixed rural/urban population.

Neonatal care is typical of secondary level “neonatal special care” [21] and includes management in incubators or under radiant heaters, respiratory support via oxygen concentrators or continuous positive airway pressure (bubble-CPAP), phototherapy, feeding support via gastric tubes and intravenous (IV) fluids, caffeine or aminophylline, phenobarbitone and broad-spectrum antibiotics. Invasive ventilation, surfactant, IV fluid pumps and continuous cardio-respiratory monitoring are unavailable. Continuous KMC was implemented as standard care during the formative trial phase in September 2017. Intermittent KMC is provided for a minimum of 60 min at periodic intervals on the neonatal unit once the neonate is off respiratory support and establishing enteral feeds. Neonates < 2000 g receive continuous KMC on an adjacent eight-bed KMC unit once they are stable in room air, are tolerating full enteral feeds and have a willing caregiver available. Neonates are transferred to the KMC unit at average 10 days of age (*n* = 148, SD 7.8) with the average KMC unit admission lasting for 6.9 days (*n* = 108, SD 4.0) and 92% (141/151) of discharged neonates attend hospital follow-up at least once, on average 7.5 days after discharge (*n* = 141, range 2–23 days) (unpublished audit data, Sept. 2017 to May 2018, H. Brotherton).

### Study population and procedures

**Fig. 1:**
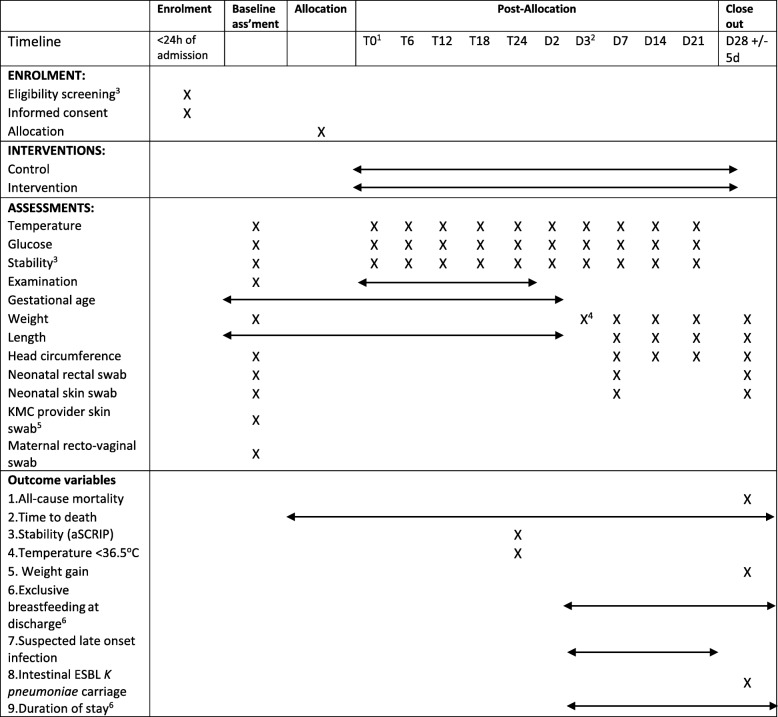
eKMC trial schedule of enrolment, interventions and assessments [16] 1. The start of study procedures (Time 0) is defined as when the pulse oximeter is attached for baseline continuous cardio-respiratory assessment, immediately prior to the intervention/control procedures commencing. 2. Participants are reviewed daily until KMC unit admission, after which they are reviewed on days 7, 14, 21, and 28 of age whilst inpatients and on day 28 as outpatients. Daily reviews are re-started if the baby is transferred back to the neonatal unit. 3. Stability definitions used during eligibility screening and routine assessments are detailed in Fig. 2. 4. Weight at 5 days of age is taken on calibrated digital scales and then is taken daily until either discharge or KMC unit admission, after which it is obtained on days 7, 14, 21, and 28 whilst an in-patient and at the day 28 follow-up if discharged. 5. Skin swab samples are taken from the first person to provide skin-to-skin contact and the mother (if different) as soon as possible and prior to any skin-to-skin contact. The relationship of the KMC provider to the participant is documented and correlated with swabs using unique, anonymised identification codes. 6. Outcomes such as feeding method and duration of stay are recorded at the time of discharge, including for participants hospitalised for > 28 days

Enrolment, interventions and assessments are outlined in Fig. 1.

**Fig. 2:**
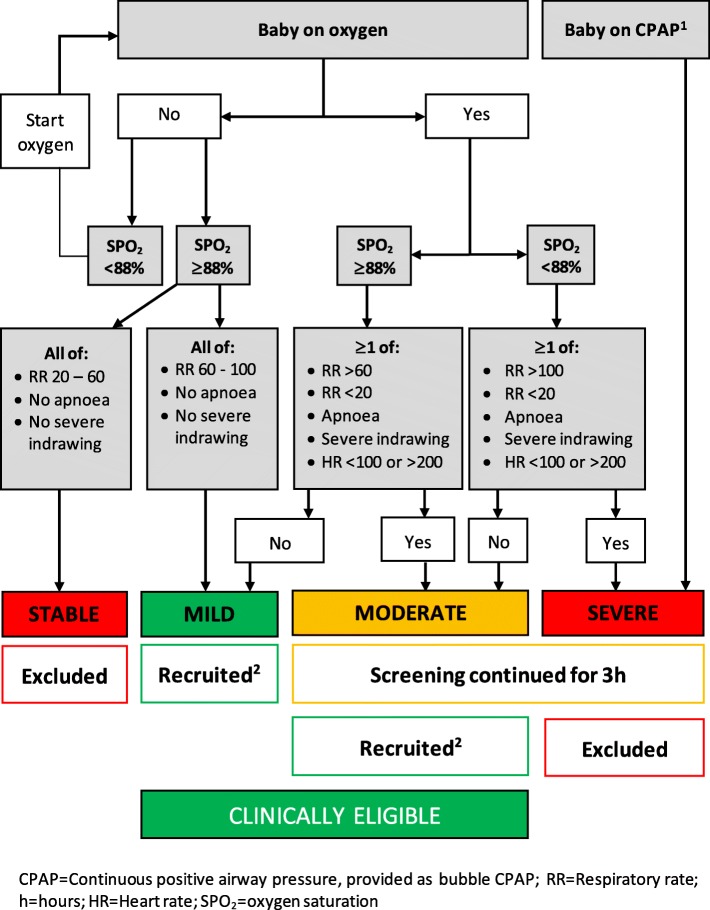
eKMC trial definitions of cardio-respiratory instability and eligibility status. 1. Criteria for starting CPAP is a Silverman-Anderson score ≥ 4 that does not improve with oxygen therapy and the absence of the following: heart rate < 100 bpm, floppy tone and seizures. 2. The neonate is recruited if a study bed is available and consent is provided by a willing caregiver

The study population is hospitalised neonates < 2000 g and age 1–24 h old at the start of the screening who meet the trial definition of mild-moderate instability based on cardio-respiratory parameters and respiratory support provision (Fig. 2).

Inclusion criteria are as follows:

- New admission of singleton or twin (inborn or out-born)
- Weight < 2000 g as per study scale
- Age 1–24 h old when screening begins
- Mother or other caregiver available and willing to provide intervention

Exclusion criteria are as follows:

- Triplets who are all admitted to the study site
- Congenital malformation not compatible with life or needing immediate surgical intervention
- Severe jaundice
- Seizures
- Stable as assessed during cardio-respiratory screening
- Severely unstable as assessed during cardio-respiratory screening or died during screening
- No study bed available
- Neonates/mothers enrolled in another research study
- No written informed consent from parent or caregiver within 24 h of admission.

### Screening for eligibility

Eligibility is assessed in all admitted neonates with referral weight ≤ 2000 g as soon as possible and once > 1 h old. Weight is confirmed using a calibrated SECA™ 757 digital weighing scale, and source documents are checked for age and other study involvement. All potentially eligible neonates aged < 24 h undergo an examination with cardio-respiratory stability assessed over 10 min using Nonin™ 2500A pulse oximeter.

Stable neonates are excluded as it is considered unethical to randomise them to a proven intervention. Mildly unstable neonates are immediately eligible for recruitment. Moderately or severely unstable neonates undergo continuous pulse oximetry with a repeat stability assessment 3 h later. Severely unstable neonates are excluded at the repeat 3 h screening, as it is not possible to provide KMC alongside resuscitation or CPAP at the study site (Fig. 2). Clinically eligible neonates are recruited if a study bed is available and a caregiver is willing to both provide the intervention (if applicable) and give written consent within 24 h of hospital admission. If eligibility criteria are met but the caregiver is only available > 3 h after the end of cardio-respiratory screening, stability is re-checked prior to consenting to avoid inadvertent recruitment of stable or severely unstable patients. Standard care under radiant heater or incubator is provided to all neonates during the screening period.

### Consent

Sensitisation activities with health workers, pregnant women and families are conducted at referral health facilities to support recruitment. Written, informed consent for participation and provision of continuous KMC (in event of randomisation to intervention arm) is sought from the first available caregiver at the study site within 24 h of admission by trained study personnel. The parent is the preferred person to provide informed consent, but other relatives may provide consent with parental informed consent being sought as soon as possible. Consent is requested in English with verbal translation into local languages using a pre-designated dictionary of definitions. Impartial witnesses are used to support the consenting process with caregivers who are unable to read or write English. Consent for obtaining and future use of paired maternal recto-vaginal and skin swab samples from the first KMC provider and mother (if different) is sought before any skin-to-skin contact occurs.

### Randomisation, allocation and blinding

An independent statistician generated a randomisation sequence using VBA (Visual Basic Application) within an Access database to produce two random number tables with stratification by admission weight categories (< 1200 g or ≥ 1200 g). Random permuted blocks of varying block sizes were used in a 1:1 allocation. The allocation sequence is concealed with sequentially numbered, opaque, sealed envelopes prepared by an independent researcher and accessible to study team only. Following the collection of baseline data, the study nurse opens the next numbered envelope for the correct weight category. The participant identifier, date and time are recorded on the outside of the envelope prior to opening, to identify any subversion of allocation sequence. Twins are allocated to the same arm, according to the first eligible twins’ weight.

Given the nature of KMC, blinding parents/caregivers and study personnel to the allocation arm and the primary outcome is not possible. Process and secondary outcome data will be anonymised, and all analyses will be blinded.

### Intervention

**Fig. 3:**
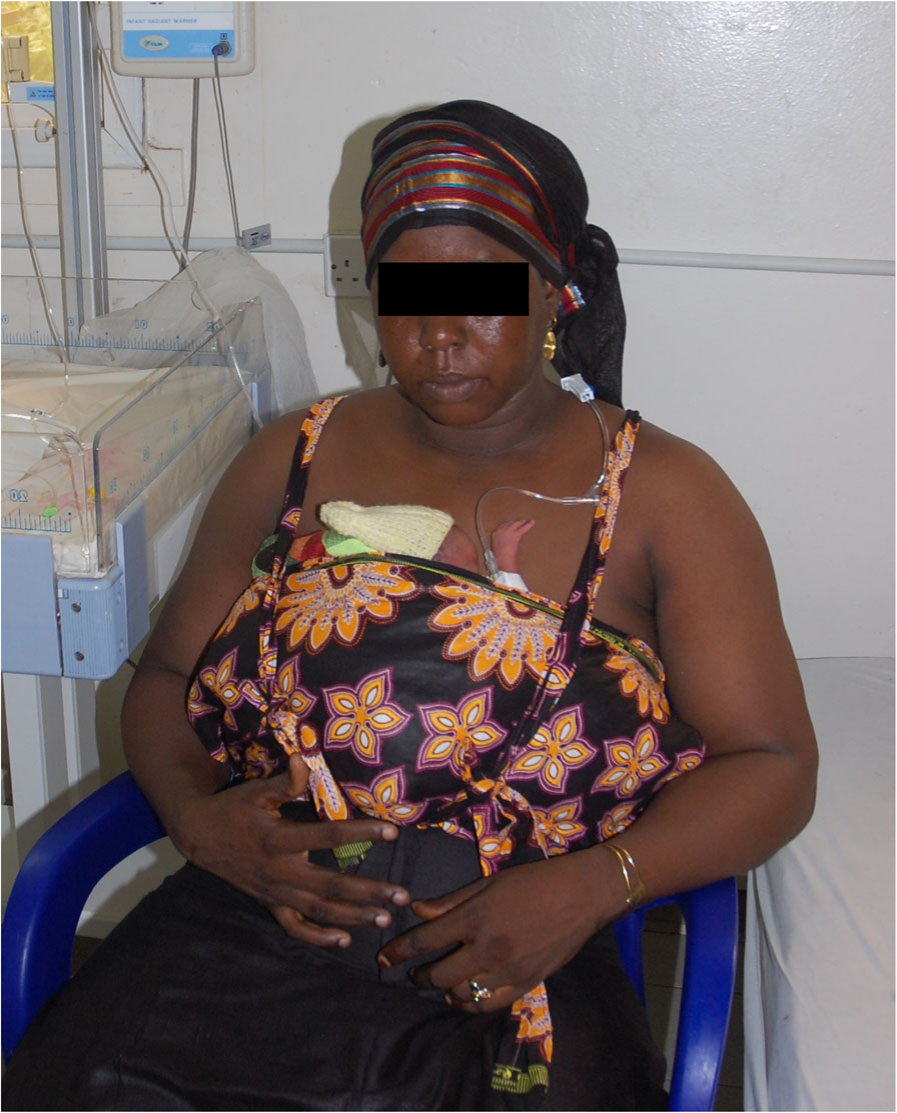
An eKMC participant receiving the intervention of ocntinuous skin-to-skin contact at the same time as other standard care treatments (H.Brotherton with caregiver consent for publication)

The terms KMC and skin-to-skin contact are used as synonyms in the literature, but the intervention under study is continuous skin-to-skin contact between neonate and caregiver started within 24 h of admission. The neonate is naked except for nappy and woollen hat and is secured with a Thari wrapper (customised KMC wrapper developed in South Africa) in a prone, frog-leg position on caregivers’ naked chest with head turned sideways (Fig. 3).

**Fig. 4:**
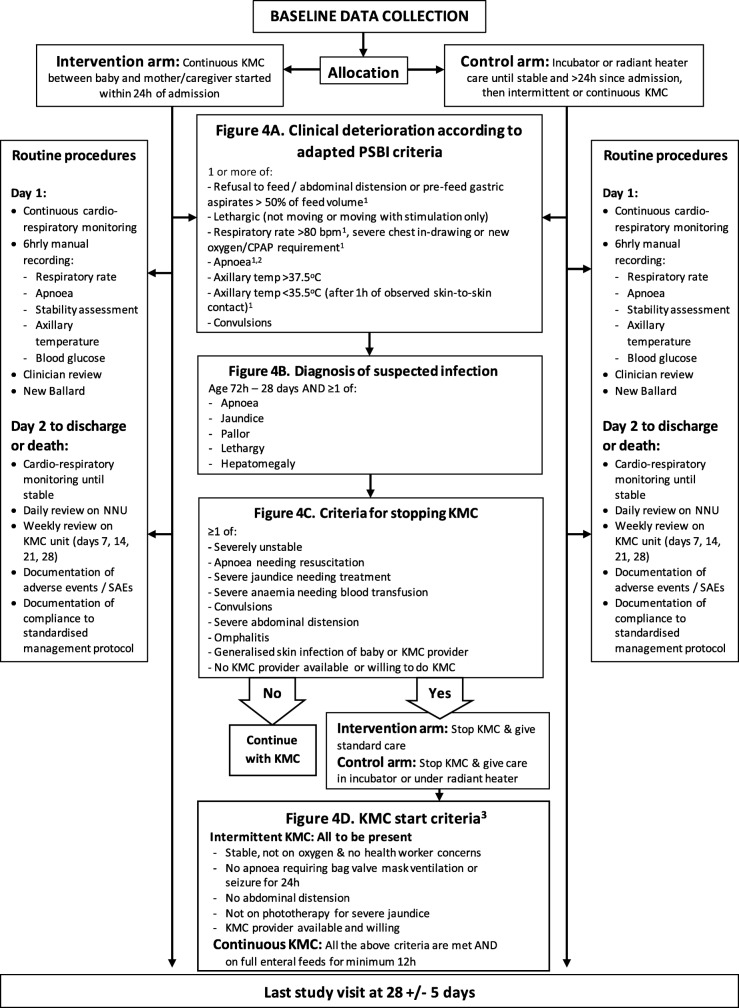
Overview of eKMC routine procedures and assessment of clinical deterioration including key trial criteria. 1. New or changed PSBI definitions to increase relevance for hospitalised preterm neonates. 2. Spontaneous apnoea with no identifiable reason, e.g., not associated with milk aspiration or end-stage respiratory failure. 3. Re-start criteria also apply to neonates in control arm at the initiation of KMC

The caregiver sits or lies down whilst the neonate receives all other treatments (oxygen via nasal prongs, intravenous (IV) fluids via peripheral venous cannula, gastric tube feeds and IV medications). If the mother is unavailable, other relatives (e.g., fathers or grandmothers) provide the intervention. KMC is advised for as long as possible, aiming for ≥ 18 h/day. When not receiving KMC, the baby remains in an incubator or under a radiant heater in the same room, with co-habitation of the radiant heater. If participants meet clinical “stopping criteria” (Fig. 4c), participants are temporarily withdrawn from the intervention arm, receive standard incubator or radiant heater care and re-start KMC once the stability criteria are met (Fig. 4d)

### Control

The neonate is managed in an incubator or under a radiant heater, naked except for a woollen hat and nappy or wrapped in a cloth. The parent/caregiver can touch, hold and feed the neonate as per standard practice but skin-to-skin contact is not provided until stability criteria are met (Fig. 4d) and after > 24 h since hospital admission. Participants then receive intermittent KMC on the neonatal unit and continuous KMC on the adjacent KMC unit (Fig. 4d).

### Flow around study site for both arms

After their baseline stability data have been collected, all participants are transferred to a “trial area” within the neonatal unit containing four small beds, chairs, incubators, radiant heater and an oxygen concentrator. This area can accommodate 8–10 patients with twin participants sharing incubators. If a neonate subsequently becomes severely unstable (Fig. 2), the affected participants are transferred to the high dependency area and then follow the standard flow around the neonatal unit. Neonates are moved from “trial area” to the KMC unit once stability criteria are met (Fig. 2), full enteral feeds have been tolerated for the previous 12 h, no phototherapy is required and both a willing caregiver and KMC unit bed are available. If participants become unwell whilst on the KMC unit, they are re-admitted to the neonatal unit and follow the standard patient flow.

### Clinical management and study procedures for neonates in both arms

Baseline anthropometric and clinical data are collected prior to randomisation with the exception of gestational age and length (within 48 h of recruitment) and socio-demographic data (within 28 days). The first available caregiver is sensitised at baseline for infection control, provision of KMC, clinical danger signs and when to call for help. All other routine and emergency treatments, including discharge, are provided according to a standardised preterm management protocol, based on pre-existing standard care at the study site and consistent with WHO guidelines. Compliance with the protocol is monitored prospectively by trial clinicians. Continuous monitoring of cardio-respiratory stability with a Nonin™ 2500A pulse oximeter occurs for a minimum 24 h of study participation, until stability is reached (Fig. 2). Direct nursing observation documents all details of the KMC provided, including the date and time of first KMC contact, relationship with the person providing KMC, KMC session frequency and duration, number of neonates receiving KMC from the same provider and the reason for not providing KMC.

Structured study reviews occur with decreasing intensity as stability improves, with reviews every 6 h for the first 24 h, daily reviews whilst on the neonatal unit and weekly reviews during the KMC unit admission (Fig. 4). The final study review at 28 ± 5 days of age occurs at EFSTH, with home visits for non-attenders. Caregivers may withdraw from the study at any time. Data collected up to the point of the most recent follow-up within 28 ± 5 days of age will be included in the analyses.

### Outcome measures

#### Primary outcome

The primary outcome is all-cause mortality at age 28 days.

#### Secondary outcomes

Secondary outcomes include the following:

1. **Time from start of study procedures to death**
  The date and time of death is recorded as soon as possible using the death certificate as a source document for in-hospital deaths and according to the caregiver verbal report for out-of-hospital deaths.
2. **Cardio-respiratory stability at 24 h of study participation (aSCRIP score)**
  The Stability of Cardio-Respiratory in Preterm Infants (SCRIP) score is an objective measure of stability used in previous KMC trials [14, 22]. The score was modified for relevance to a pre-stabilised preterm population receiving oxygen (Additional file 2).
3. **Prevalence of hypothermia (axillary temperature < 36.5 **^**o**^**C) at 24 h of study participation**
  Axillary temperature is measured with an electronic thermometer as the average of three consecutive values.
4. **Proportion of neonates exclusively breastfeeding at the time of discharge**
  Exclusive breast-feeding and use of formula milk are recorded prospectively by direct observation and questioning of caregiver at time of discharge.
5. **Mean daily weight gain at age 28  ± 5 days (g/day)**
  This gain is the difference in weight between baseline and day 28 ± 5 days, as measured on a calibrated study scale.
6. **Incidence of clinically suspected infection from 3 to 28 days of age or latest follow-up**
  In the absence of a standardised clinical definition for infection in preterm neonates, a two-step process is used to identify clinically suspected infection (Fig. 4a & b). The WHO’s Possible Serious Bacterial Infection (PSBI) criteria [23] were adapted to increase the relevance to a hospitalised preterm population receiving KMC (Fig. 4a). If any aPSBI criteria are present, a clinician examines the baby for features of suspected infection [24] (Fig. 4b), and blood ± cerebro-spinal fluid (CSF) cultures are obtained if these criteria are met. *BACTEC Peds* Plus™/F vials are inoculated with minimum 1 ml venous blood by study clinicians and processed as soon as possible within 24 h in an automated Bactec® 9050 BD machine at MRCG at LSHTM. Samples with positive signal undergo sub-culture as per standard culture methods, species identification by API 80 system and antibiotic susceptibility testing by disc diffusion according to CLSI 2017 guidelines. CSF samples are collected by study clinicians as soon as possible and in the absence of contra-indications. CSF is transported to MRCG laboratories at room temperature within 1 h of collection for routine microbiological and biochemical analysis.
  Isolation of clinically significant bacteria are recorded, with coagulase negative staph (CONS) and bacillus species predefined as non-pathogenic. A secondary analysis of the effect of KMC on confirmed (culture positive) infection is planned.
7. **Prevalence of neonatal intestinal carriage of extended-spectrum beta-lactamase (ESBL)-producing *Klebsiella pneumoniae*at age 28 ± 5 days**
  Rectal swabs are taken with size appropriate FLOQ™ swabs and stored for batch microbiological processing. Additional paired maternal and/or caregiver-neonatal carriage flocked swab samples obtained at baseline, 7 days (neonatal) and 28 ± 5 days (neonatal) (Fig. 1) are stored for future microbiological and molecular processing.
8. **Mean duration of stay (hours)**
  Time from study site admission to discharge is documented prospectively according to source documents for the first admission episode. This information indicates if a participant is discharged after 28 days of age.

#### Other variables of interest

Adverse events (e.g., abnormal blood glucose, jaundice, apnoea) are observed in both arms as safety parameters. The number, proportion and reason for temporary withdrawal from the intervention arm is recorded. Weekly anthropometry (weight, length and head circumference) provides additional indicators of growth. Continuous heart rate and oxygen saturation measurements alongside 6-hourly aSCRIP scores (Additional file 2) are recorded for the first 24 h of study participation for a planned secondary analysis of cardio-respiratory stability.

### Data collection, management and security

All study personnel are trained in ICH-GCP, study objectives and study-specific procedures, in addition to being trained in clinical newborn care and KMC. Socio-demographic, clinical and summary laboratory data are collected using the REDCap™ data entry system with built-in range and consistency checks. Length is obtained with a Seca210 measuring mat and head circumference with non-stretchable tape measures using triplicate measures and regular inter- and intra-observer standardisation checks with double-blind assessments against clinician assessment. Vital signs are measured over 10-min periods to generate mean values, using calibrated Nonin™ 2500A pulse oximeters for heart rate and oxygen saturation with manual recording of respiratory rate. Gestational age assessment is done by trained clinicians using the New Ballard [25] score with regular inter-observer variability monitoring. All biological samples are processed or stored (maximum -70 °C) at MRCG at LSHTM laboratories and biobank (ISO 15189 Accredited), including paired neonatal-caregiver carriage swab samples and invasive isolates intended for future exploration of infection mechanisms. Cardio-respiratory stability data from Nonin™ 2500A pulse oximeters is downloaded, analysed with NVision™ software and reconciled with the study database. The daily dose of KMC is automatically calculated before reconciliation with the study database. All data are securely stored on a MRCG central server or at the study site with restricted access. A non-identifiable unique study number for neonate and caregiver is used to maintain confidentiality for all data, including stored samples, with linkage of neonatal and caregiver identifications.

### Sample size

A total of 392 subjects (1:1 ratio) is required to detect a 30% relative reduction in the primary outcome (power 80%, alpha = 0.05) with recruitment planned for 2 years. This number is based on an expected mortality rate of 48% [20], with adjustment for an estimated 15% reduction in mortality due to trial implementation. Loss to follow-up rates are expected to be low (< 3%) due to the restricted geographical area, co-ordination of follow-up with routine appointments and re-imbursement of travel expenses.

### Statistical analyses

A detailed statistical analysis plan will be made available at the trial registry before analysis commences. Analysis of all outcomes will be on an intention-to-treat basis. Since complete twin allocations account for an estimated 20% of the study population and are independent risk factors for mortality [26], adjustment for twin correlation will be undertaken using linear mixed effects models for continuous data and generalised estimating equations for binary data.

#### Comparability of participants in two arms

Baseline characteristics will be presented by the allocation arm using descriptive statistics. Key indicators of standard hospital care received will be compared for both arms at baseline and during admission.

#### Flow of participants

**Fig. 5:**
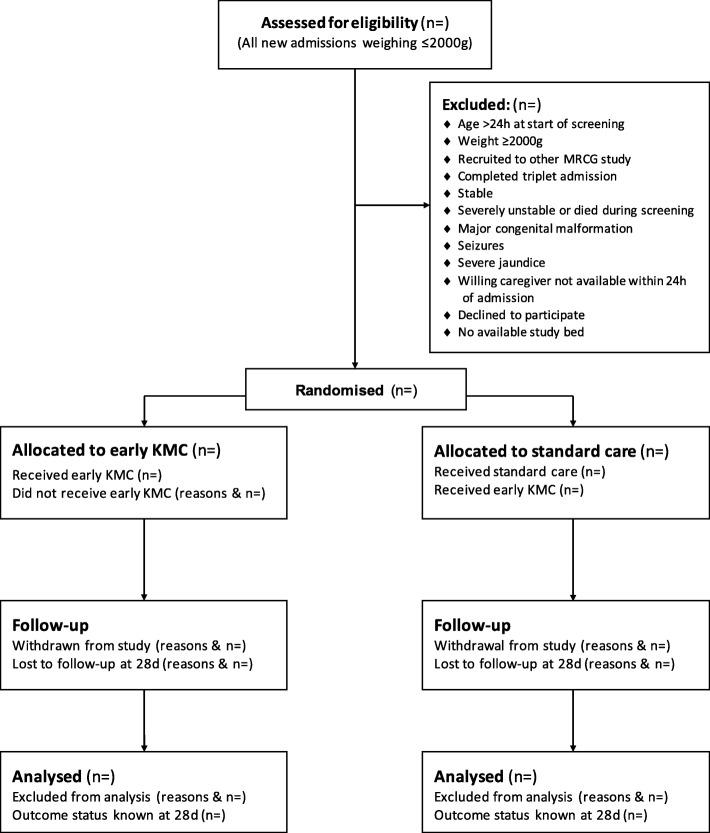
Trial flow diagram, as per CONSORT guidelines 2010 [27]

The number and flow of subjects through screening, randomisation, allocation, follow-up and analysis will be documented, as per CONSORT 2010 guidelines [27], with reasons for exclusion, withdrawal and non-analysis being described (Fig. 5). Participants will be excluded from the final analysis if they have been permanently withdrawn.

#### Primary and secondary outcome analysis

The number of subjects with the primary outcome will be calculated for each arm and generalised estimating equations used to calculate risk ratios and the number needed to treat with confidence intervals. Analysis of secondary outcomes will be performed according to the type of data and using either number of subjects or person time as the denominator. Continuous variables will be compared between arms using random effects models, and categorical data with generalised estimating equations. Survival analysis of the time to death within first 28 days after birth will be performed using cox regression with frailty. In the event of multiple events for the same participant (e.g., infection), each episode will be considered an isolated event.

Missing data are expected to be few and will be addressed with a complete case analysis. Sub-group analyses for all outcomes will be performed according to birth weight categories (< 1200 g; ≥ 1200 g) and multiple birth. Tests for effect modification by weight and multiple birth will be performed. The following will be calculated for both arms as indicators of adherence: mean chronological age at first KMC contact, mean time since admission at first KMC contact, daily dose of KMC (hours per study day) and average daily dose of in-patient KMC (per number of days admitted from enrolment). A sensitivity analysis of all outcomes will be performed according to average in-patient daily dose of KMC.

### Safety reporting and study monitoring against ICH-GCP standards

Adverse events are any clinical event resulting in a change in management of the participant after enrolment and until age 28 days. Serious adverse events (SAE) are defined as death, life-threatening events (e.g., apnoea requiring bag-valve-mask ventilation, or severe instability), events carrying a risk of permanent or temporary disability (e.g., suspected meningitis), re-hospitalisation within 28 days of age and prolonged hospitalisation for ≥ 28 days.

A local safety monitor, the sponsor and the trial monitors are informed of all SAEs within 24 h of the study team being aware with a detailed report sent within 2 working days for fatal and 5 days for a non-fatal SAE. All fatal SAEs are reported to the ethics committees monthly and within 7 days if related to the intervention. Non-fatal SAE’s are communicated to the ethics committees annually or within 14 days if related to the intervention. A Data Safety Monitoring Board (DSMB) receives a bi-monthly safety report with bi-annual meetings to monitor recruitment, progress and safety. DSMB members include the clinical trialist/statistician (chair), a neonatologist experienced in a similar setting, a West African clinical trialist and an independent statistician. An un-blinded interim analysis will be conducted after randomisation of 50% of target sample size with pre-specified stopping rules for efficacy, using the Haybittle-Peto rule [28, 29] and will inform recommendations to the Trial Steering Committee (TSC), who will make the final decision on study continuation. Study procedures and documents are monitored for compliance to ICH-GCP standards by MRCG monitors every 3–6 months, with auditing determined by the sponsor.

## Discussion

Evaluating the effect of continuous KMC before full stabilisation is a global research priority, stated by WHO [6] with the potential to contribute towards reducing the unacceptably high global neonatal mortality, enabling progress towards the neonatal mortality target SDG3.2 by 2030, as well as promoting a family-centred approach to newborn care. eKMC is one of the first trials to address this evidence gap and is expected to provide robust evidence in addition to novel mechanistic insights, particularly regarding infections, which are one of the major pathways to mortality for preterm neonates.

KMC reduces severe infections (6.6% vs 13.1%, RR = 0.5, 95% CI 0.36–0.69) and nosocomial infections (4% vs 11%, RR = 0.35, 95% CI 0.22–0.54) with intermittent or continuous KMC in stable neonates [6]. However, previous KMC trials have lacked clear case definitions for infection and a paucity of microbiologically confirmed data are available from resource-limited settings [6]. eKMC will contribute towards understanding the infection prevention effects of KMC by using a validated nosocomial risk score [24] microbiological testing and exploration of impact on carriage of antimicrobial resistant bacteria.

During eKMC trial piloting, we identified important challenges, which are outlined below with mitigating approaches:

- Challenge 1 - Recruitment: The unavailability of caregivers willing to consent *and* provide the intervention within 24 h after birth is a major recruitment barrier due to high rates of maternal illness or post-caesarean section and absence of other family members at the hospital during the early admission period. Sensitisation activities with pregnant women and their families and health workers are undertaken at referral centres to encourage recruitment. A high proportion of either severe instability or death occurs before or during the screening process, reflecting the high proportion of out-born neonates and a vulnerable population. Access to sufficient study beds for the intervention was a limiting factor, and the number of study beds was increased from 2 to 4 during the piloting period to facilitate recruitment.
- Challenge 2 - Non-blinded trial: KMC could not be blinded for the family or researchers. Selection and allocation bias are prevented through rigorous screening and randomisation procedures with objective stability markers, and transparent reporting of non-recruitment will be performed. Treatment bias is minimised via a protocolised approach to standard care with prospective monitoring of adherence, comparable clinical monitoring and caregiver education for both arms.
- Challenge 3 - Twins: Like much of West Africa, the twin birth rate in The Gambia is high at 16.7/1000 live-births [30] with greater risk of premature delivery and neonatal death compared to singletons [26, 30]. Evaluation of the intervention in twins is essential for generalisability of results and to target the most vulnerable neonates. Investigators anticipate that 30% of participants will be twin gestation, with complete twin enrolment accounting for 20% of the study population. This may lead to differences in provision of the intervention in addition to independently impacting the trial outcomes. All efforts to adjust for multiple births will be made during analysis.
- Challenge 4 – Improvements to standard care leading to potential dilution of the intervention effect and risk of inadequate power: Alongside externally driven improvements to newborn care at the study site, eKMC implementation has resulted in major improvements to standard care for both trial and non-trial neonates. In collaboration with the hospital, the Gambian Government Ministry of Health and Social Welfare and UNICEF The Gambia, an eight-bed KMC unit was established, and continuous KMC was embedded in standard care in 2017. A protocolised approach to standard care of preterm neonates was also introduced at the site to reduce the risk of treatment bias. Although highly beneficial from an individual patient perspective, these improvements in care are expected to reduce both the power of detecting a difference in the primary outcome and may reduce differences between allocation arms, diluting the intervention effect. These changes to standard care will be explored in a linked process evaluation, based on the MRC guidance for evaluation of complex interventions [31] and using data collected before and after trial implementation. Activities will include a survival analysis of neonatal case fatality rates using published data from the study site [20] and prospective data collection for all admissions over the trial period, tracking of the changes made to standard newborn care, and KMC implementation progress monitoring [32].

If early KMC for pre-stabilised neonates is shown to be beneficial, we need to understand how to implement in a real-world setting. eKMC-generated implementation and safety data will be valuable, particularly when combined with similar trials, such as the multi-site OMWaNA trial in Uganda [33] and WHO-led multi-centre I-KMC trial [34]. We aim to align data definitions and maximise opportunities for pooled analyses with the OMWaNA trial.

The primary outcome results of the eKMC trial will contribute to the global evidence base for use of KMC before stabilisation in preterm neonates, with secondary outcome results and other analyses providing insights to how KMC is effective, particularly regarding infection prevention. The eKMC trial aims to inform one of the great divides between resource-limited and resource-rich settings and improve the chance for newborns everywhere to survive and thrive.

## Supplementary information

**Additional file 1.** SPIRIT 2013 Checklist: Recommended items to address in a clinical trial protocol and related documents.

**Additional file 2.** aSCRIP definition.

## Data Availability

The final trial dataset will be available on requests made to the PI or institutional delegate. The results of this study will be published in an open access format in a peer-reviewed biomedical journal, in addition to the PI’s doctoral thesis. The results will be disseminated at relevant international scientific forums and communicated to the World Health Organisation. The Gambian Government Ministry of Health and Social Welfare and other relevant local stakeholders and participant families will be directly informed of the study results.

## Abbreviations

AE: Adverse events
aPSBI: Adapted possible severe bacterial infection
aSCRIP: Adapted stability of cardio-respiratory in preterm infants
CI: Confidence interval
CLSI: Clinical and Laboratory Standards Institute
CONS: Coagulase-negative staphylococcus
CPAP: Continuous positive airway pressure
CSF: Cerebral-spinal fluid
DSMB: Data safety monitoring board
EFSTH: Edward Francis Small Teaching Hospital
ESBL: Extended-spectrum beta lactamase
ISO: International Organisation for Standardisation
IV: Intravenous
KMC: Kangaroo mother care
LSHTM: London School of Hygiene & Tropical Medicine
MRCG: Medical Research Council Unit The Gambia at London School of Hygiene & Tropical Medicine
NMR: Neonatal mortality rate
PI: Principal investigator
RCT: Randomised controlled trial
RR: Risk ratio
SAE: Serious adverse event
SD: Standard deviation
SDGs: Sustainable development goals
TSC: Trial Steering Committee
VBA: Visual Basic Application
WHO: World Health Organisation
