# Supplementary material for: Protocol for a randomised trial of early kangaroo mother care compared to standard care on survival of pre-stabilised preterm neonates in The Gambia (eKMC)
Source: Trials. 2020 Mar 6;21:247. doi: 10.1186/s13063-020-4149-y (PMC7059319; doi:10.1186/s13063-020-4149-y)
Supplement: Supplementary file 2 — Additional file 2. aSCRIP definition. [file 13063_2020_4149_MOESM2_ESM.pdf]

		2 points	1 point	0 points
A	**Cardiovascular stability**:<br><br>Heart rate<br>(beats per minute)	120 – 160 for 10 mins continuously	100 - 120<br>or<br>161 – 200 for >5 out of 10 mins	<100<br>or<br>>200 continuously for >5 out of 10 mins
B	**Respiratory stability:**<br><br>Breathing pattern and respiratory rate (RR)<br>(breaths per minute)	Regular breathing<br><br>And/or<br><br>RR 20 – 60 continuously for 10 mins	Irregular or periodic breathing with 1 or more pauses for <10 secs<br><br>And / or<br><br>RR 20 – 29 or 61 – 100 for >5 out of 10 mins	Irregular breathing with 1 or more pauses for >10 secs<br><br>And / or<br><br>Irregular breathing with 1 or more pauses for <10 seconds but which result in oxygen saturation <88%<br><br>Need for bag-valve-mask ventilation<br><br>And / or<br><br>RR <20 or >100 for >5 out of 10 mins
C	**Oxygen saturation (%)**	>88% continuously in room air for 10 mins	>88% continuously for >5 out of 10 mins in oxygen	<88% continuously for >5 out of 10 mins despite oxygen

Stable = 6 points; Mild – moderately unstable = 3 – 5 points; Severely unstable = 0 – 2 points
